# Spider Silk Constructs Enhance Axonal Regeneration and Remyelination in Long Nerve Defects in Sheep

**DOI:** 10.1371/journal.pone.0016990

**Published:** 2011-02-25

**Authors:** Christine Radtke, Christina Allmeling, Karl-Heinz Waldmann, Kerstin Reimers, Kerstin Thies, Henning C. Schenk, Anja Hillmer, Merlin Guggenheim, Gudrun Brandes, Peter M. Vogt

**Affiliations:** 1 Department of Plastic, Hand and Reconstructive Surgery, Hannover Medical School, Hannover, Germany; 2 Clinic for Pigs, Small Ruminants, Forensic Medicine and Ambulatory Service, University of Veterinary Medicine Hannover, Hannover, Germany; 3 Department of Small Animal Medicine and Surgery, University of Veterinary Medicine Hanover, Hannover, Germany; 4 Department of Plastic and Reconstructive Surgery, Department of Surgery, University Hospital Zurich, Zurich, Switzerland; 5 Institute of Cell Biology in the Center of Anatomy, Hannover Medical School, Hannover, Germany; Université de Technologie de Compiègne, France

## Abstract

**Background:**

Surgical reapposition of peripheral nerve results in some axonal regeneration and functional recovery, but the clinical outcome in long distance nerve defects is disappointing and research continues to utilize further interventional approaches to optimize functional recovery. We describe the use of nerve constructs consisting of decellularized vein grafts filled with spider silk fibers as a guiding material to bridge a 6.0 cm tibial nerve defect in adult sheep.

**Methodology/Principal Findings:**

The nerve constructs were compared to autologous nerve grafts. Regeneration was evaluated for clinical, electrophysiological and histological outcome. Electrophysiological recordings were obtained at 6 months and 10 months post surgery in each group. Ten months later, the nerves were removed and prepared for immunostaining, electrophysiological and electron microscopy. Immunostaining for sodium channel (NaV 1.6) was used to define nodes of Ranvier on regenerated axons in combination with anti-S100 and neurofilament. Anti-S100 was used to identify Schwann cells. Axons regenerated through the constructs and were myelinated indicating migration of Schwann cells into the constructs. Nodes of Ranvier between myelin segments were observed and identified by intense sodium channel (NaV 1.6) staining on the regenerated axons. There was no significant difference in electrophysiological results between control autologous experimental and construct implantation indicating that our construct are an effective alternative to autologous nerve transplantation.

**Conclusions/Significance:**

This study demonstrates that spider silk enhances Schwann cell migration, axonal regrowth and remyelination including electrophysiological recovery in a long-distance peripheral nerve gap model resulting in functional recovery. This improvement in nerve regeneration could have significant clinical implications for reconstructive nerve surgery.

## Introduction

The development of effective treatments for peripheral nerve injury with a substance defect injury is of considerable medical interest. Severe trauma, tumor infiltration or neuroma resection can cause severe peripheral nerve damage and can lead to permanent destruction or even complete functional deficit of the injured extremity. At the moment, gold standard is the surgical intervention with nerve suturing or when required nerve transplantation. But often the results are still disappointing and methods to improve functional outcome are desirable and necessary. A special challenge regarding nerve repair are long distant nerve defects.

A promising alternative to conventional grafting is the use of artificial nerve grafts, of which the Integra Neurosciences Type I collagen sheath (NeuraGen™, Integra Neuroscience; Plainsboro, NJ, USA) is commercially available for treating short nerve defects clinically. Artificial nerve grafts are usually in the form of nerve tubes and conduits [1], [2]. Clinical trials demonstrated that tubular repair of 3–5 mm nerve gaps with silicone tubes yielded functional recovery at least as good as routine microsurgical repair [3], [4].

Although artificial nerve grafts constructed from non-resorbable materials (e.g. conduits made from silicone or polyethylene) have yielded some degree of functional recovery, long-term complications often mean that a second surgical procedure is necessary to remove the conduits. These may actually become detrimental by virtue of toxicity or tendency to constrict the nerve [1]. A nerve graft made of bioresorbable materials is thus a promising alternative for promoting successful nerve regeneration. A variety of resorbable materials have been examined for nerve tissue engineering applications, including two classes of polymer. One class consists of natural polymers such as chitosan and alginate [5]–[7]. Another class is formed from synthetic polymers such as polyglycolic acid (PGA), poly L-lactic acid (PLLA), poly-3-hydroxybutyrate (PHB) and their copolymers or derivatives [8]–[10]. Most promising for optimization of nerve tissue engineering is the production of biomimetic nerve guidance channels, providing chemotactic, topological, and haptotactic signalling to cells by surface functionalization with cell binding domains and the use of internal-oriented matrices/fibres with possible release of neurotrophic factors [11]. Several biocompatible and biodegradable materials have been studied for guide for nerve defect repair. These materials include poly(caprolactone) (PCL), a biosynthetic blend between PCL and chitosan (CS) and a synthesised poly(ester-urethane) (PU) with natural polymers such as gelatin (G), poly(L-lysine) (PL) and blends between chitosan and gelatin (CS/G) as internal coatings for nerve guides [12]. However, translational approaches to clinical application is limited and it seems that several aspects, microsurgery, cell and tissue transplantation, material science and gene transfer have to be combined to archive optimal results [13].

Although axonal regeneration in long nerve gaps, in which most experiments describing defects up to 40 mm in rodent models, can be observed, recovery of function may be insufficient if the axons fail to reach target. Various in vivo tests were carried out for the repair of small (5 mm), medium (15 mm) and long (45 mm) peripheral nerve size defects in rodents. Especially, mechanical stiffness of PCL and the lack of flexibility of the guide to rat movements impaired the repair of 45 mm-long defects in 8-month period [14].

The usage of spider silk fibers as guidance material for nerve regeneration is a novel and unique idea. The spider dragline silk from the spider *Nephila clavipes* has crucial unique characteristics regarding nerve regeneration guidance: the spider dragline silk can be easily harvested and silk fibres are generally long-term degradable by a proteolytic degradation induced by a foreign body response. The tensile strength of the spider silk is up to 4.8 GPa (comparable to Kevlar 49), the lightweight 1.3 g/cm^3^, toughness and elasticity is up to 35%, and it is thermally stable up to ∼250°C [15], [16]. In vitro experiments demonstrated remarkable adherence of Schwann cells to the spider silk with cell proliferation [17]. Constructs consisting of decellularized vein grafts filled with spider silk as nerve guidance material showed complete biodegeneration over time without challenging tissue reaction [18]. These spider silk constructs have been used as an artificial nerve conduit to promote Schwann cell growth and regeneration of sciatic nerves in a rat model of peripheral nerve injury and repair [18]. The efficacy of nerve constructs consisting of decellularized veins filled with spider silk fibers as guidance material in large animal nerve injury models and in long distance defects (>5.0 cm) has not been studied.

The present study addresses the issue of potentially enhancing peripheral nerve regeneration in long distance nerve defects in a large animal model.

## Results

### Macroscopic appearance

**Figure 1:**
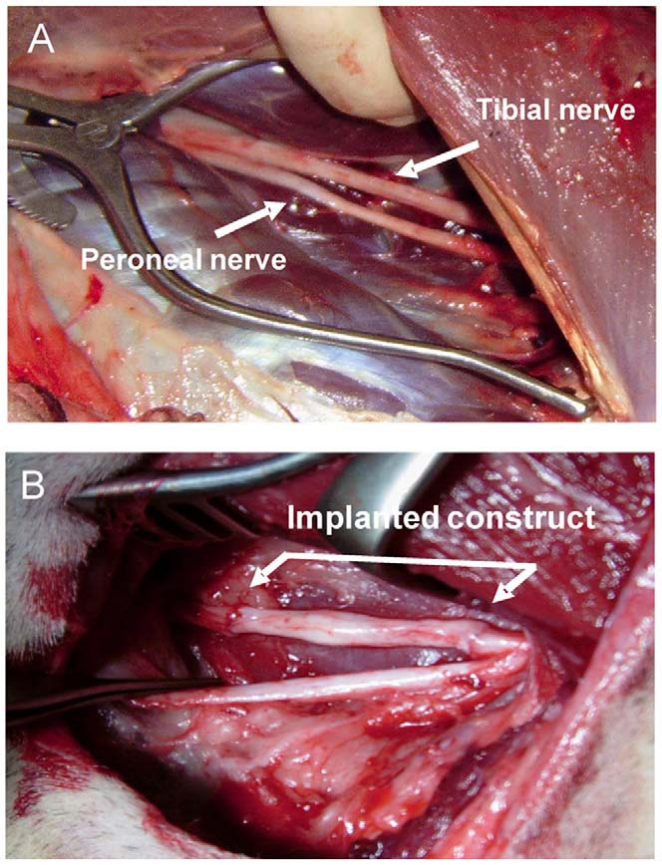
Demonstration of lesion side and nerve defect induction of peripheral nerve and construct implantation in adult sheep. (A) showing tibial and peroneal nerve (arrow head). (B) Nerve defect in tibial nerve of 6 cm and bridging of nerve defect lesion with vein/spider silk construct which is sutured between proximal and distal nerve stump of the 6 cm nerve defect in adult sheep.

In the adult sheep, tibial and peroneal nerve are easily accessible (Fig. 1A) by surgical intervention and chosen as lesion model. As lesion model, nerve defects of 6 cm were induced by surgical resection of tibial nerve and resulting tibial nerve defect, in which either autologous nerve grafts or spider silk constructs (Fig. 1B) were implanted for bridging the nerve lesion site to guide axonal regeneration. Following surgical intervention, all sheep in both groups showed no obvious signs of systemic or regional inflammation. To assess the impact of construct transplantation on functional outcome, all animals were tested using sensitivity of hind limb. Normal gait was defined as flat unguligrade stepping with a clear impression of the full ventral surface of the hoof at regular stride intervals. In all animals, immediately following nerve transection, the limb became profoundly paretic, showed poor limb flexion, the hock joint was overflexed, the fetlock was partially flexed, and absent postural reactions and hypoalgesia of the limb from distal of the stifle were recognized. As recovery occurs (21±5 days), gait improves in both groups, in the sham control and the construct transplant groups; animals began to stand with their hind limb plantar surfaces on the operated side touching the ground at three weeks post-operatively and walked with ease at two months post-operatively. Four months after surgery, the spider silk grafted sheep could stand upright on both hind limbs, and coordinated stepping of the hind limbs was observed during walking. No obvious differences in limb strength were observed between the operated limb and unoperated limb. The calf muscle groups of the operated limb showed some discrete atrophy.

### Graft survival and integration into sutured nerve followed by endogenous Schwann cell invasion

**Figure 2:**
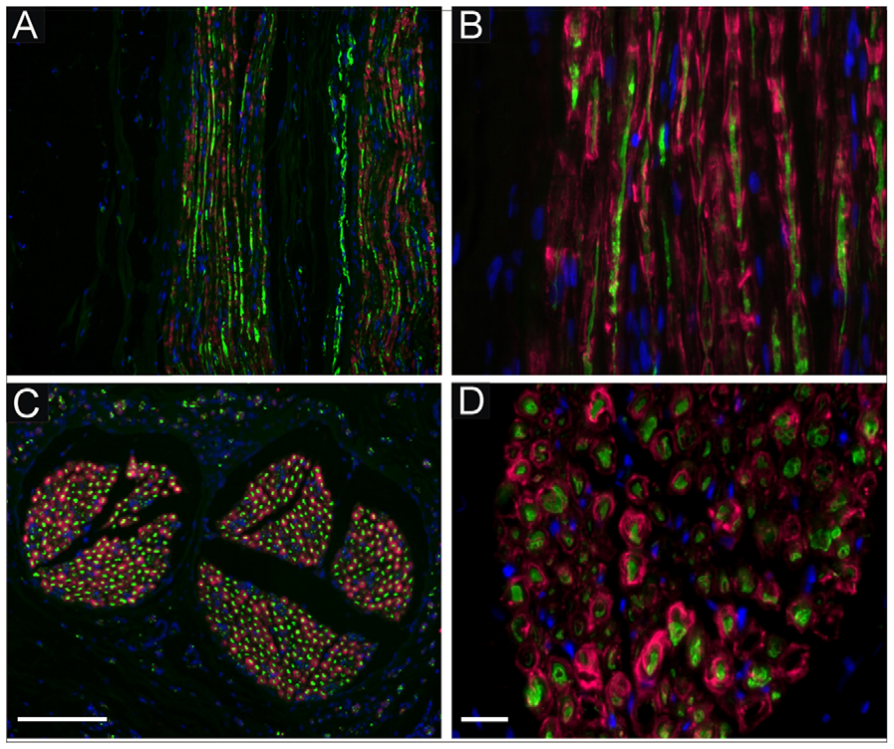
Histological analysis of regenerated fibers following spider silk construct implantation for nerve defect bridging. Immunostaining of regenerated nerve sections with neurofilament (NF, A–D in green) for regenerated axons and S100 for identification Schwann cells (A–D in red) demonstrating that axons regenerated throughout the construct and endogenous Schwann cells migrated into the construct (A and B, red). Cell nuclei are stained with DAPI (blue). Cross sectioning of regenerated nerve fibers by implantation of spider silk construct showed immunopositive staining for S100 and co-localization of neurofilament revealed ensheathment of regenerated axons by endogenous Schwann indicative for remyelination of regenerated nerve fibers in the construct (C and D). Scale bar in C = 10 µm, scale bar in D = 8 µm.

Ten months following surgery, the animals were euthanized and the nerves removed and frozen sections were prepared for immunostaining procedures. In both, the control group (autologous nerve transplantation, data not shown) and the spider silk construct transplantation group, neurofilament (NF) immunostaining for axons indicated that axons regenerated across the suture repair site and into the nerve grafts bridging the entire length of the defect. Axons were longitudinally aligned throughout the entire length of the regenerated lesion site. Note that even fascicle structure of nerves was reestablished in regenerated nerve segments and that endogenous Schwann cells (SCs) are present within regenerated nerves in the spider silk construct group (Fig. 2A–D) as identified in the nerve by S100 staining (Fig. 2A–D, red). The SCs in the nerve construct group arose from migration as the grafts were deficient in SCs. In longitudinal sections of the regenerated nerve, SCs alignment was in close approximation with the regenerated axons and cross sections of the nerves showed that SCs were wrapped around regenerated axons indicating that endogenous migrated SCs not only migrated and survived in the construct, but also functionally ensheathed regenerated axons. The cells were positioned on the NF labeled axons (green) in the typical position of myelinating Schwann cells [19].

### Improved axonal regeneration of myelinated nerve fibers

**Figure 3:**
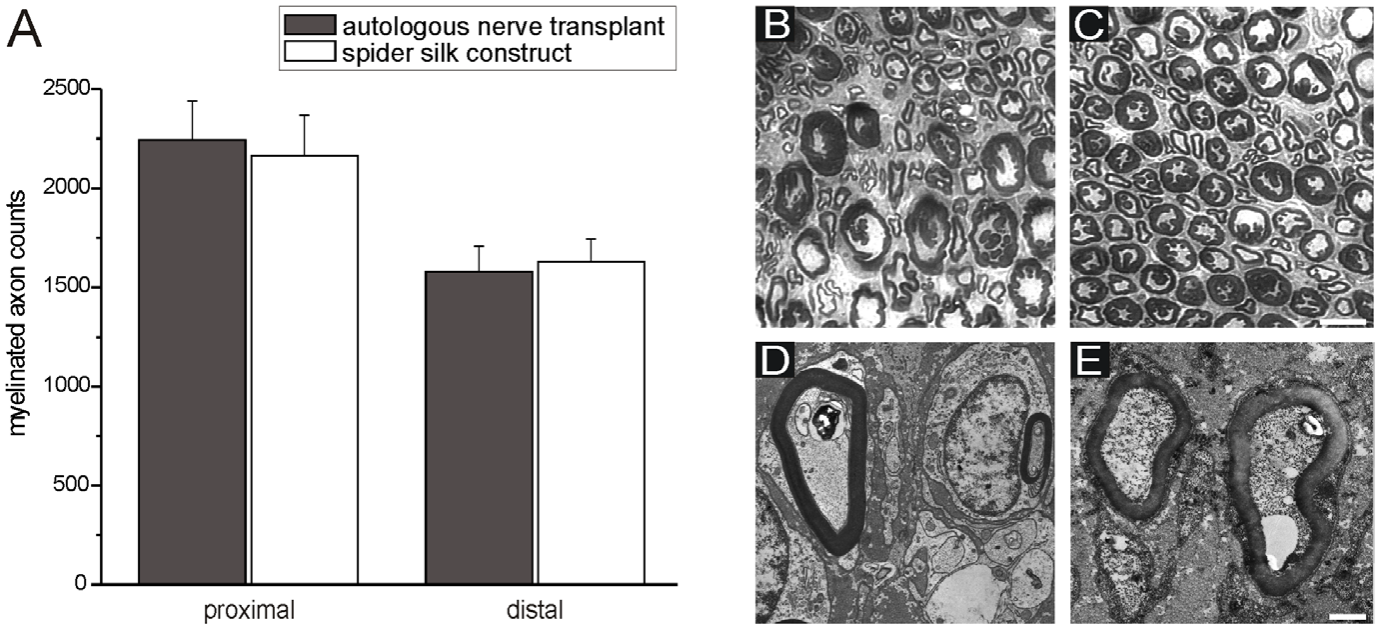
Axon counts and demonstration of myelinated of myelinated profiles proximal and distal to either the autologous transplanted nerve or the implanted spider silk construct. (A) Axon counts lesion site and at regions proximal and distal were compared between both groups. Plastic embedded coronal sections of autologous nerve transplant (B and D) and spider silk construct (C and E) at 10 months post surgery. The improved organization of myelinated axons after transplantation of the spider silk construct could be demonstrated by light (C) and transmission electron microscope (E). All spider fibers were degraded in a subtle manner, since an inflammation has never been seen. Scale bar for C and D in D = 3 µm, for E and F in F = 1 µm.

To analyze the extent of axonal regeneration, transverse sections of autologous control and construct transplanted nerves were obtained and photographed ten months after surgery. Myelinated axon counts were performed. Transplantation of autologous nerve transplantation or spider silk construct resulted in axonal regeneration followed by myelination and preservation of myelinated axons proximal and distal to the repair site. In the autologous control nerves, axon counts of regenerated and myelinated fibers were 2245±194 proximal to the repair site and 1578±129 distal to the repair site. In the construct implanted transplantation group, there were 2163±205 axons proximal to the repair site and 1630±114 distal to the repair site. A summary of these results is presented in Fig. 3. There was no statistical significance observed between axon count results if autologous and construct transplant groups proximally and distally. Photomicrographs distal (2 mm) to suture repair site for autologous control and construct transplantation groups are shown in Fig. 3B and C. Note the greater myelinated axon organization in distal sections in the construct transplant group (Fig. 3C). This can also be demonstrated in the transmission electron microscope for (Fig. 3D and E). The myelinated axons were separated from each other only by collagen fibrils and some capillaries. After ten months no silk fibers are leaved behind. The degradation process was subtle, because no inflammation, infiltration of immune cells or proliferation of phagocytes has been found anywhere.

### Formation of nodes of Ranvier with sodium channel expression

**Figure 4:**
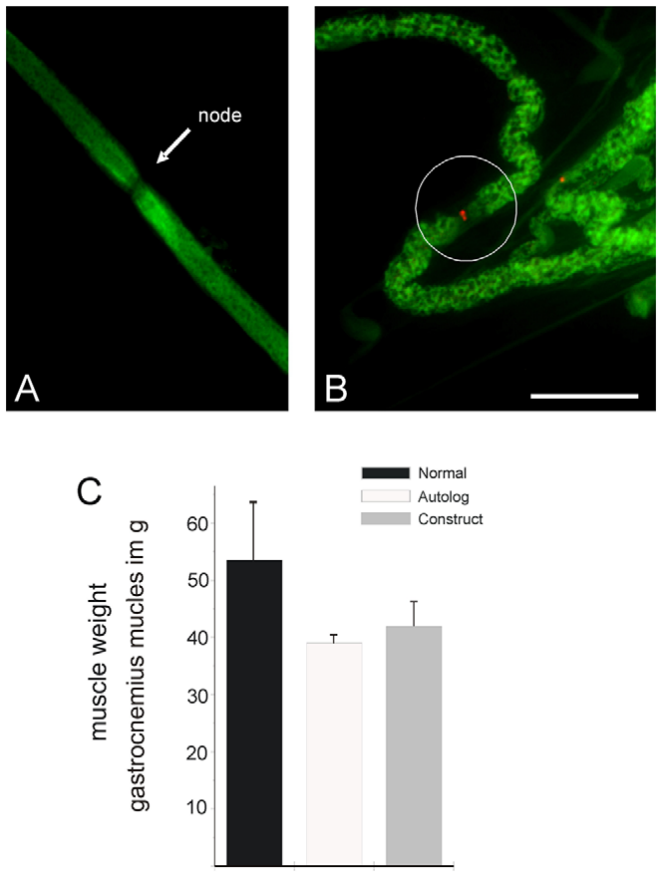
Demonstration of typical nodes of Ranvier on regenerated axons. (A) The nodes showed high density of sodium channel NaV1.6 (B) Determination of muscle weight after injury and regeneration of tibial nerve defect by either autologous nerve transplant or spider silk construct implantation in comparison to normal muscle weight of uninjured gastrocnemius muscle in adult sheep (C). Scale bar in B = 6 µm.

Nodes of Ranvier were observed on the regenerated axons within the nerve grafts as “breaks” in myelin staining between SC segments. A putative node of Ranvier is indicated in a single regenerated axon within the regenerated and myelinated axons within the construct (Fig. 4A). Immunostaining for the voltage-gated sodium channel NaV 1.6 demonstrated high expression of this sodium channel on the axon at these gap regions indicating the nodal nature of these regions of axon membrane and that appropriate sodium channel subtype is present on the regenerated axons (Fig. 4B).

### Morphological and morphometric examinations of the gastrocnemius muscle

The gastrocnemius muscle started to atrophy after sciatic nerve injury, and the size of the gastrocnemius muscle on the operated side decreased in sheep of both the autograft and construct grafted groups. Atrophy was qualitatively observed from physical examination, and quantitatively from weight comparison between normal and the operated groups including the autologous and construct implanted animals: gastrocnemius weight reduction could be observed in the transplant group contrast to uninjured control group, but no statistically significant differences in weight of the muscle were measured between autologous and construct implanted animals (Fig. 4C) ten months after surgery and nerve defect regeneration. This is in accordance to the axon counts where the spider silk construct resulted in comparable myelinated axon counts as the autologous nerve transplant as described in Figure 3A–C.

### *In vivo* electrophysiological recordings

**Figure 5:**
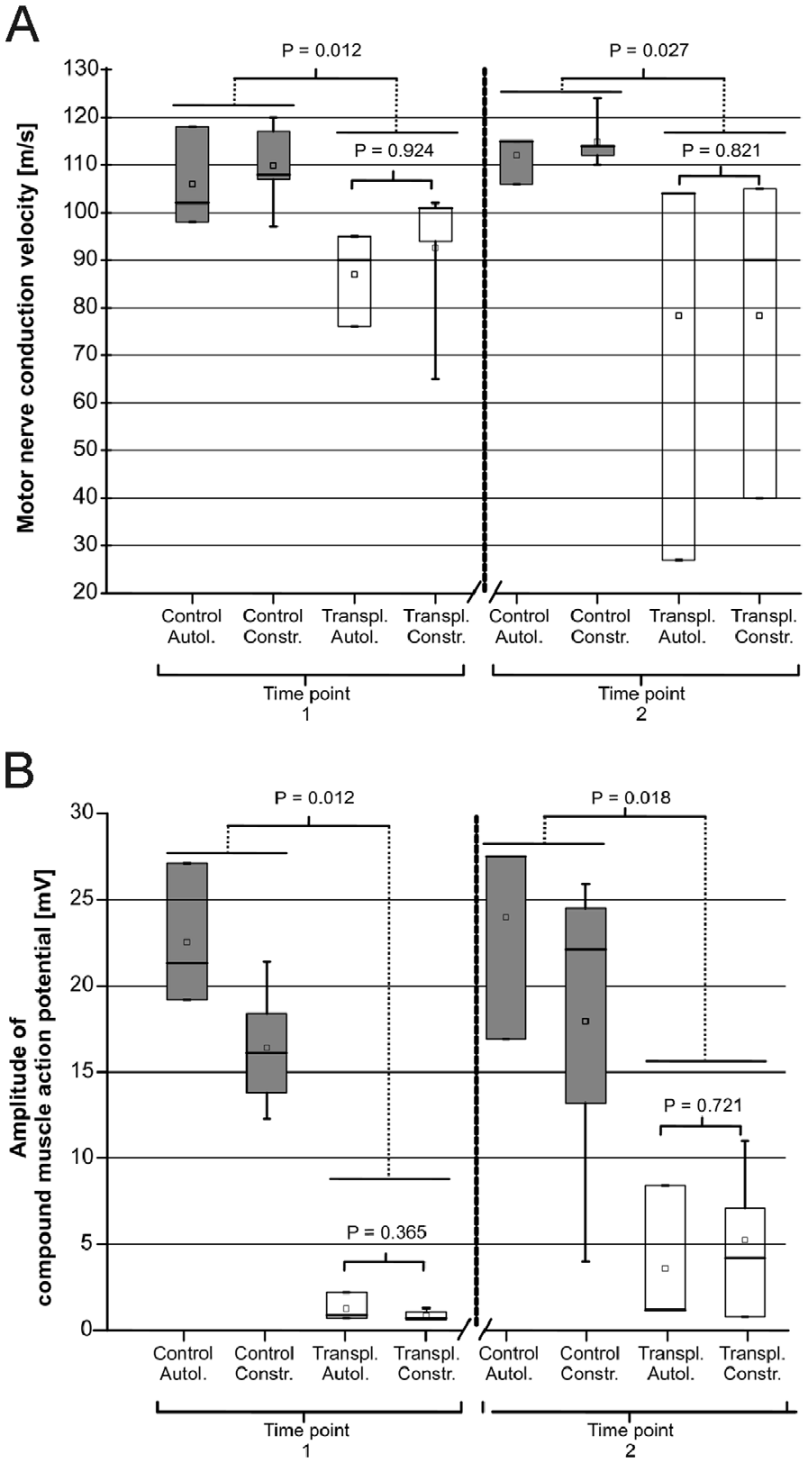
Electrophysiological recordings of spider silk constructs and autologous nerve transfer. (A) EDxNCV: Box plot diagram of the in vivo measurement of the motor nerve conduction velocity (mNCV) at time point T1 (after 6 months post surgery) and at time point T2 (after 10 months post surgery). ⊤ =  maximum, ⊥ =  minimum, — =  median, □ = mean. A box includes 50% of the values. P values of Mann–Whitney–Wilcoxon-test for paired samples are indicated in the figure. Values of P≤0.05 were considered significant. (B) EDxAMP: Box plot diagram of the in vivo measurement of the compound muscle action potential amplitudes at time point T1 and at time point T2. ⊤ =  maximum, ⊥ =  minimum, — =  median, □ = mean. A box includes 50% of the values. P values of Mann–Whitney–Wilcoxon-test for paired samples are indicated in the figure. Values of P≤0.05 were considered significant.

**Figure 6:**
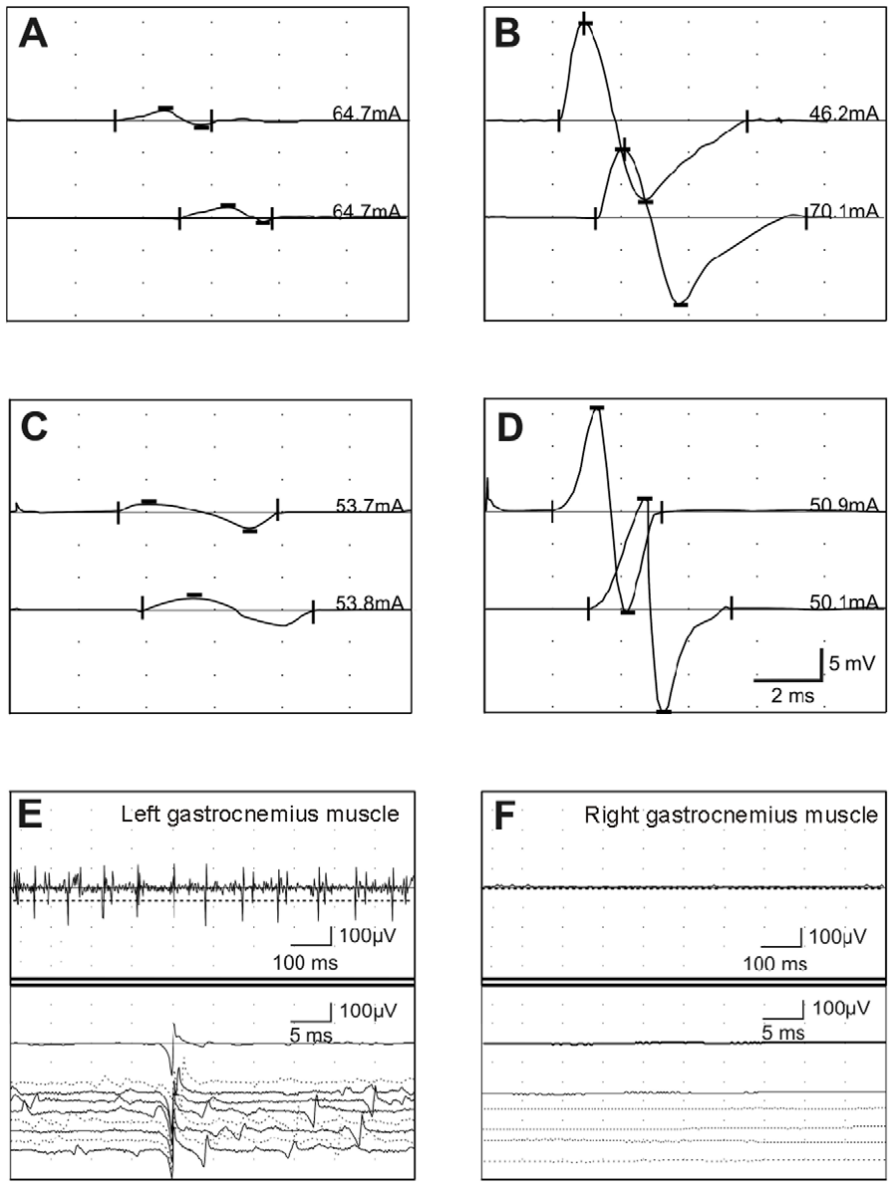
Representative electrophysiological recordings of spider silk constructs and autologous nerve transfer. (A–D) Electroneurography traces at time point 1 (6 months post-surgery) of the left (A and C) or right tibial (B and D) nerve. (A) CMAP's of left tibial nerve; autologous nerve transfer; (B) corresponding CMAP's of the right control tibial nerve (C) CMAP's of left tibial nerve with spider silk construct; (D) corresponding CMAP's of right/control tibial nerve. The upper traces in each panel are after distal stimulation and the lower traces after proximal stimulation. The calibrations in D correspond to A–D. Stimulus current is indicated next to each trace. E. Electromyographic recordings at time point 1 (6 months post surgery) of left gastrocnemius muscle (autologous nerve transfer) (F). Corresponding electromyographic control recordings of right gastrocnemic muscle from the same animal. Note the fibrillation potentials and positive sharp waves in (E).

Electrophysiological recordings (electromyography (EMG) and electroneurography (ENG)) were obtained at 2 different time points (T1 = 6 months and T2 = 10 months post surgery) in each control nerves (n = 8), and autologous (n = 3) and construct (n = 5) engrafted groups. Electrophysiological recordings of spider silk constructs and autologous nerve transfer are shown in Figure 5 in box plot diagrams Representative original traces of these recordings are presented in Figure 6.

Electrophysiological recordings were obtained at 2 different time points (T1 = 6 months and T2 = 10 months post surgery) in each control nerves (n = 8), and autologous (n = 3) and construct (n = 5) engrafted groups. The motor nerve conduction velocity (mNCV) and the amplitude of the compound motor action potential (CMAP) for the three groups are summarized in Figure 5A and B. At time point T1 the mNCV of the autologous control group (n = 3) ranged from 98.0 to 118.0 m/s with the median at 102.0 m/s; the mNCV range of the construct control group (n = 5) was 97.0–120.0 m/s, the median 112.0 m/s respectively. These values are in accordance with the literature [20]. The range of the mNCV from the autologous transplanted group (n = 3) was 76.0–95.0 m/s with the median at 90.0 m/s; the mNCV of the construct transplanted group n = 5) ranged from 65.0 to 102.0 m/s with the median at 97.5 m/s.

At time point T2 the mNCV of the autologous control group (n = 3) ranged from 106.0 m/s to 115.0 m/s with the median at 115.0 m/s; the mNCV range of the construct control group (n = 5) was 110.0–124.0 m/s with the median at 114.0 m/s. The range of the mNCV from the autologous transplanted group (n = 3) was 27.0–104.0 m/s with the median at 104.0 m/s; the mNCV of the construct transplanted group (n = 4) ranged from 40.0 m/s to 105.0 m/s with the median at 90.0 m/s (one nerve in this group was not measurably, because of tearing at the proximal nerve suture).

At time point T1 the amplitude of the autologous control group (n = 3) ranged from 19.2 to 27.1 mV with the median at 21.3 mV; the amplitude range of the construct control group (n = 5) was 12.3–21.4 mV, the median 16.1 mV respectively. The range of the amplitude from the autologous transplanted group (n = 3) was 0.7–2.2 mV with the median at 0.9 mV; the amplitude of the construct transplanted group (n = 5) ranged from 0.6 to 1.3 mV with the median at 0.7 mV.

The measurement of the amplitude of the autologous control group at the second time point revealed values (n = 3) ranging from 16.9 mV to 27.5 mV with the median at 27.5 mV; the amplitude range of the construct control group (n = 5) was 13.2–25.9 mV with the median at 22.1 mV. The range of the amplitude from the autologous transplanted group (n = 3) was 1.2–8.4 mV with the median at 1.2 mV; the amplitude of the construct transplanted group (n = 4) ranged from 0.8 mV to 11.0 mV with the median at 4.6 mV (one nerve in this group was not measurably, because of tearing out at the proximal nerve suture).

At both time points the mNCV of the transplanted nerves (autologous and construct group) was significant lower than of the respective control nerves (T1: P = 0.012; T2: P = 0.027). Also the statistical comparison of the CMAP amplitudes of control to transplanted nerves (autologous and construct group) reveals a significant difference (T1: P = 0.012; t2: P = 0.018). Statistical comparison for differences of the amplitude or the mNCV between the autologous and construct group revealed no significant distinction between the groups at the 2 time points (mNCV at T1: P = ;0.924; mNCV at T2: P = 0.821; amplitude at t1: P = 0.365; amplitude at t2: P = 0.721). Statistical comparison between the amplitude from time point T1 to T2, for mNCV did not demonstrate a significant difference. No significant difference indicative of an enhanced/declined myelination or difference in conduction parameters were found between the autologous and the construct groups. Also there was no statistical evidence for an improvement of evaluated parameters from time point T1 to T2 for these groups.

Development of transplantation complications like proximal tearing of the transplanted construct in one sheep at time point T2 were readily identified with the electrophysiological evaluation by an absence of CMAP at proximal stimulation (proven by adjacent surgical exploration). In this mentioned sheep, a neuroma formed at the T2 time point which was been suspected based on the electrodiagnostic evaluation, because of a drop of the mNCV from 90.0 m/s at the first measurement to 27.0 m/s.

The ENG traces (Figure 6 A to D) demonstrate a similar appearance with temporal dispersion, prolonged onset latencies and reduction of the amplitude of the compound motor action potential (CMAP) for autologous graft (Fig. 6A) and spider silk construct (Fig. 6B). ENG recordings from the control (right) temporal dispersion and prolonged onset latencies resulted in a reduced motor nerve conduction velocity (mNCV). Electromyography (EMG) revealed spontaneous pathological activity (fibrillation potentials and positive sharp waves) in all examined left side gastrocnemius muscles for both the autologous or construct groups. In all right side gastrocnemius muscles the EMG was normal (Figure 6 E, F). EMG data were not further analyzed.

## Discussion

Recent advances in nerve tissue engineering have greatly promoted the generation of nerve conduits, which may be implanted empty, or may be filled with growth factors, cells or fibres [21]. It is worth mentioning that longitudinal biomaterial filaments have also been introduced into a nerve conduit to facilitate nerve regeneration across extended nerve defects [3], [9], [22]. Accordingly, multi-component complex nerve guides are often referred to as ‘tissue engineered nerve grafts’.

In this study, the artificial nerve grafts were used to bridge long-distance defects of peripheral nerves in adult sheep. The regenerated axons were found ten months after lesion induction and nerve repair and moreover, the axons were as well myelinated by endogenous invaded Schwann cells. These results demonstrate that our artificial nerve constructs consisting of acellularized veins filled with spider silk results in peripheral nerve regeneration similar to autologous nerve transplantation. Additionally to these morphological results, electrophysiological in vivo measurements and the clinical observations of the improved locomotor function of the sheep proved on a functional level the comparable performance of the artificial nerve constructs to autologous nerve transplantation.

In vivo evaluations of the CMAP and the mNCV as quantifiable functional parameters allow an estimate of axonal growth and myelination respectively. Monitoring of regeneration itself, maturation and maintenance of regenerated motor axons is achieved by this technique in various studies [23]. The CMAP amplitude reflects the number of functional axons, while the CMAP latency and the dependent mNCV are determined by the degree of myelination [24]. The statistical analysis of these parameters (Figure 5 A, B and Figure 6) between the two groups was not able to show significant differences in the regeneration progress on a functional level. This evidence is proven and complemented by morphological results of this study.

Moreover we were able to show that functional monitoring by electrophysiological recordings is a non invasive method to detect implantation failures in a large animal model, as shown in the case of neuroma formation in one sheep of this study. Such in vivo examinations seem to be very useful for continuous evaluation of the regeneration process or of side effects.

In contrast to CMAP measurements and the determination of the mNCV the collected EMG data did not reveal auxiliary information beyond the already clinical visible evidence of tibial nerve paresis on the operated sides in both groups. For this reason further analysis of this data was not accomplished.

The precise mechanism responsible for functional recovery after implantation of our spider silk constructs in experimental models is not fully understood. Several mechanisms including axonal sparing, sprouting and plasticity associated with novel polysynaptic pathways, recruitment of endogenous SCs and remyelination have been suggested to contribute to improvement in function in large nerve defects bridged with guidance material for nerve regeneration. In both groups of regenerated nerves, after autologous nerve as well as spider silk construct transplantation, viable SCs could be observed after ten months indicating the endogenous migration and invasion of SC into the implant. Importantly, the newly formed nodes of Ranvier of the regenerated axons expressed sodium channel subtype NaV1.6 [25], the normal predominant nodal sodium channel. This indicates that engraftment of endogenous Schwann cells into injured nerve can reconstitute myelin and appropriate sodium channel organization necessary for proper impulse conduction.

The spider silk itself is slowly absorbed in vivo. According to the published literature, the rate of absorption is dependent upon the implantation site, mechanical environment, and variables related to the health and physiological status and the diameter of the silk. Furthermore, alterations in silk processing may cause conformational changes in the protein structure potentially increasing or decreasing susceptibility to degradation. Regardless, silk protein fibers will degrade in vivo; rates are variable depending on the factors listed above [26]. In our own studies no traces of residual spider silk could be detected in ultrastructural investigations and the degradation process must be subtle, because no signs of inflammation were present.

Several studies published in the recent literature were concerned about the potential application of silk fibroin fibres derived from *Bombyx mori* silk. Yang et al. used oriented silk fibroin filaments successfully to guide nerve regeneration along a 10 mm gap in a sciatic nerve defect model in rats achieving nearly as good results as autologous nerve transfer [27]. The use of silk fibroin proteins, however, is a basically different approach to the use of spider silk as technically processed material is used instead of native biomaterial. This difference stand for advantages as for disadvantages as well as the processing allows for technical adaption like tube formation by gel spinning [28] or combination with other materials [29] but might also be more reliant on modifications to enhance cytocompatibility than native spider silk [30].

Questions arise in terms of the potential translation of these experimental findings to a clinical setting. It is certainly advantageous to avoid donor side morbidity from harvesting a nerve for transplantation. Another problem is the availability of donor nerve tissue. In large brachial plexus injuries for example, where multiple nerve transplants might be necessary, the limitation of available autologous nerves for transplantation is hindering optimal outcome, it has to be triaged which function is most important. With a nerve bridging material resulting in similar regeneration and remyelination as the gold standard autologous nerve transplantation these limitations would be finally resolved. Most results described in previous studies have shown peripheral nerve repair with nerve conduits in nerve defects with 2 cm or less substance defects. The challenge is to bridge larger nerve substance defects. Here, we could demonstrate in an extensive nerve defect model that with our spider silk/vein construct axonal regeneration in a large animal model is successful. The applied electrophysiological measurements emerge as a useful non invasive method for monitoring progress of peripheral nerve repair and for detection of aberrant conditions which might develop (e.g. neuroma formation) in a clinical setting.

Further studies may enhance results obtained by implantation of our artificial nerve graft construct, optimize the support provided by the axotomized neurons and denervated distal stumps, and protect the target muscle so that may be even faster or functionally better regeneration is archived in contrast to autologous nerve transplantation. Moreover, transplantation of autologous SCs or even olfactory ensheathing cells (OECs) could in principle assist more rapidly the regenerative process, but still have to be investigated in the presented context.

For example, if severed nerve is surgically reapposed it may take time for the endogenous SCs to appropriated differentiate and organize to provide an optimal regenerative environment. Cultured SCs or OECs transplanted to the apposition site could facilitate the regenerative process [19], [25], [31]. The beneficial effect of silk filaments on axonal guiding has also recently been described by Madduri et al., who described a method for electrospinning of Bombyx mori silk fibres with constant release of neurotrophic factors, e.g. GDNF and NGF, over four weeks [32]. In their findings they describe axonal outgrowth on their nanostructured elements along with glial cell proliferation and migration when culturing embryonic dorsal root and spinal cord explants on their constructs. A comparable approach was intended by Uebersax et al. who used air-dried or freeze-dried silk fibroin films for biofunctionalization with NGF [33]. Biofunctionalization by neurotrophic factors for optimized nerve repair might also be a future option for the use of spider silk filaments in the context of peripheral defects.

While we raise issues in terms of potential problems which need to be overcome before translation to a clinical study using their approach, this is not to detract from the importance of these experimental findings. Clearly, there is a strong need for establishing reliable methods for nerve substance defects in clinical practice.

## Materials and Methods

### Preparation of acellularized venules

Animals were kept in accordance with the guidelines of the German Animal Welfare Act. The protocol was approved by a review committee of the state of Lower Saxony, Germany. Venules were taken from veins of the lower extremities of pigs with weight of 50 kg. The venules had a diameter of approximately 3–4 mm and a length of 6 cm. The venules were excised and freed from adherent fat. Afterwards veins were washed in PBS (phosphate-buffered saline, Biochrome, Berlin, Germany) and incubated in trypsin/EDTA for 24 hrs (Biochrome, Berlin, Germany). The procedure was repeated after washing with PBS for two weeks. Then the venules were washed with 0.1% tert-octyphenyl-polyoxyethylen (Triton X-100, BioRad, Munich, Germany) for 3 h. All steps were conducted at 37°C under continuous shaking. After extensive washing the venules were histologically controlled by hematoxilin-eosin and trichrome stainings and frozen at −80°C until usage.

### Spider silk harvest

Spider silk fibres were collected from adult females of the genus *Nephila* kept at our local animal facility. For experimental practice we used the major-ampullate-dragline, which serves the spider as security rope and building material. The dragline silk is secreted by the major ampullate glands and exits from spigots on the anterior lateral spinnerets. The spiders were gently fixated with a compress. We mechanically pulled out the fibres with a spider silk winding machine after stimulation of the major ampullate gland and collected them on spools. On average, we gained 150 m length of silk per hour. The spider silk was sterilized and consequently instantly prepared for usage.

### Construct fabrication

The spider silk was cut to the appropriate length and pulled through the acellularized venules until filling out one quarter of the venule diameter. The constructs were kept in Hanks balanced salt solution (HBSS, Biochrome Germany) and engrafted at the same day.

### Autologous nerve transplantation and implantation of construct

Experiments were performed in accordance with the German Animal welfare guidelines for the care and use of laboratory animals. The Hannover Medical School and the Nds. Landesamt für Verbraucherschutz und Lebensmittelsicherheit approved all animal protocols. 24 adult sheep (50–80 kg) were used for this experiment. The sheep were anesthetized with xylazine (xylazin 2%, CP Pharma, Burgdorf, Germany) with a dosage of 0.1 mg/kg and ketamine (ketamin 10, Selectavet, Weyern Holzolling, Germany) with a dosage of 4 mg/kg intravenously applied. Anaesthesia was maintained using ketamine bolus injections of 2 mg/kg i.v. The sciatic nerves were exposed near the piriformis tendon and a nerve defect was induced over a distance of 6 cm. In 12 animals the nerve was switched from proximal to distal and resutured into the nerve as autologous transplant, in 12 animals the spider silk/vein construct was placed by microsutures into the defect.

### Nerve harvest and histology

Sciatic nerves from both groups and untreated contralateral sites were processed for immunohistochemistry as described previously after 10 months. Briefly, the tissue was removed, washed with PBS and then with ice-cold 4% paraformaldehyde in 0.14 M Sorensen's phosphate buffer, pH 7.4. Sciatic nerves were excised and placed in fresh fixative to achieve a total fixation time of 25 min. Tissue was rinsed several times with PBS and cryoprotected in 30% sucrose in PBS overnight at 4°C. Ten micrometer cryosections of the sciatic nerve were cut and mounted on glass (Fisher Superfrost Plus, Fisher Scientific, Inc. Waltham, Mass, USA) slides. The sections were processed for double immunofluorescent staining for detection of S100, sodium channel NaV1.6, or neurofilament (NF). The primary antibodies used were as follows: polyclonal NaV1.6 (1∶100; Alomone Labs, Jerusalem, Israel), monoclonal NF (1∶1000; Sigma-Aldrich, St. Louis, MO, USA) and monoclonal S100 (1∶300, Dako, Carpinteria, California, USA) Secondary antibodies used were as follows: goat anti-rabbit IgG-Cy3 (1∶2000; Amersham Biosciences, Piscataway, NJ, USA) and goat anti-mouse IgG-Alexa Fluor 633 or Alexa Fluor 546 (1∶1000; Invitrogen, Eugene, OR, USA).Sections were examined with conventional fluorescence microscopy (Zeiss, Oberkochen, Germany).

### Plastic embedding and morphometric analysis for axon counting

Sciatic nerves were excised, stored overnight in fixative (2% paraformaldehyde plus 2% glutaraldehyde in 0.14 M Sorensen's phosphate buffer, pH 7.3), cut into 2 mm segments, notched to indicate orientation, post-fixed with 1% osmium (Polysciences, Warrington, PA) for 4 hr, dehydrated, and embedded in Epox−812 (Ernest F. Fullam, Latham, NY) using standard plastic embedding protocols. Semithin sections (1 µm) were collected of plastic embedded tissue and counterstained with methylene blue and azure II (0.5% each in 0.5% borax) to identify areas of interest.

To assess the extent of axonal regeneration, axon counts of myelinated profiles within the transection site and at regions proximal and distal were be compared between construct-transplanted group and autologous nerve transplanted controls. Quantitative morphometric analysis of remyelination was conducted by sampling remyelinated axon density in step sections collected every 0.25 mm along the rostral caudal axis of the nerves and integrating across distance essentially as described previously [34].

Axon counts and cross-sectional area measurements were obtained 10.0 mm rostral and caudal to the midpoint of the lesion using a Nikon Microphot micoroscope (100x) and image analysis software (Bioquaont Novaprime). The number of axons at these regions was compared between autologous and construct transplanted groups. One-way ANOVA was conducted, followed by a Dunnett's test to identify specific pairwise differences between the means. Comparison analyses were conducted using SPSS version 10.1.3.

For the ultrastructural analysis thin sections (70 nm) on Formvar-coated copper grids were stained with saturated uranyl acetate and lead citrate and analysed in the Zeiss 902 transmission electron microscope. Representative micrographs were arranged with Adobe Photoshop 5.0.

### *In vivo* electrophysiological recordings

In a second set of experiments, animals (n = 8) were anesthetized according to the formerly described protocol 6 months and 10 months after peripheral nerve lesion and transplantation. All stimulations of the control and operated tibial nerves and electroneurographic (ENG) recordings of the compound muscle action potentials (CMAP) in the gastrocnemius muscles were performed, using a Vicking Quest electrodiagnostic device (Nicolet Viking Quest IV, Nicolet EBE GmbH, Kleinostheim, Germany). The tibial nerves of each side were stimulated supramaximally with a rectangular stimulation-impulse. The strength of the stimulation ranged between 20–80 mA. This stimulation was applied with a frequency of 1.0 Hz and duration of 0.1 ms to evoke a CMAP in the distally located gastrocnemius muscle. Stimulation electrodes had a diameter of 0.5 mm, a length of 7.5 cm and were coated with Teflon® (Part No. 019–411500, Nicolet EBE GmbH, Kleinostheim). The tibial nerve was stimulated distally caudally to the popliteal fossa and proximally in the trochanteric fossa between the greater trochanter and the tuber ischiadicum. The ground electrode was placed between the distal stimulation point and the recording electrode. For recording the CMAP a bipolar concentric needle electrode with a diameter of 0.6 mm and a length of 60 cm was used (Part No. 019–721700, Nicolet EBE GmbH, Kleinostheim). The CMAPs were displayed and stored for the calculation of the mNCV and determination of the amplitude on the electrodiagnostic device. Amplitudes were measured from peak to peak and for determination of the latency also the first negative peak was used. Calculation of the motor nerve conduction velocity (mNCV) was performed automatically by the electrodiagnostic device using the following formula and the rectally measured body temperature as correction factor:
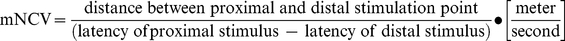



All results are shown as range and median. Data was analysed by a Shapiro-Wilk test for Gaussian distribution and according to results of analysis non-parametric testing by Mann–Whitney–Wilcoxon-test for paired samples was performed to compare the amplitudes and the mNCV of control and operated tibial nerves intra-individually. Statistical analysis was performed using commercial available software (Origin v7, OriginLab Corporation, One Roundhouse Plaza, Suite 303, Northampton, MA 01060, USA). Values of P≤0.05 were considered significant.

Additionally to the ENG recordings of the CMAP the bipolar recording electrode was used to perform a needle electromyography (EMG) of tibial nerve innervated muscles (e.g. gastrocnemius muscle) on each side of the pelvic limbs under the same anaesthesia at the two time points. Original traces were stored in the electrodiagnostic device.
